# Vaccine storage and stock management practices in Vihiga County, Kenya

**DOI:** 10.1080/20523211.2024.2337128

**Published:** 2024-04-17

**Authors:** Eclayne Naswa Wanyonyi, Evans Sagwa, Stany Banzimana, Domina Asingizwe

**Affiliations:** a EAC Regional Centre of Excellence for Vaccines, Immunization, and Health Supply Chain Management, College of Medicine and Health Sciences, University of Rwanda, Kigali, Rwanda; b Ministry of Health, Nairobi, Kenya

**Keywords:** Vaccines, effective vaccine management, practices, Kenya

## Abstract

**Background:**

Effective vaccine management is crucial to maintain vaccine potency. To achieve this, elements, such as temperature management, stock management, infrastructure, cold chain equipment and waste management, need to be properly implemented. Therefore, this study was conducted to assess the vaccine storage and stock management practices in public health facilities within Vihiga County.

**Methods:**

A descriptive cross-sectional design was used. Eighty-six public health facilities were selected and one staff involved in handling vaccines from each facility participated in the study. The study utilised survey questionnaires and observational checklists to collect data.

**Results:**

All facilities visited use standard vaccine requisition forms for ordering and receiving vaccines and physical stock counts are done in all facilities. The majority of immunising healthcare workers knew how to condition icepacks 88.4%; however, 57.0% did not know all the heat-, cold- and light-sensitive vaccines. Status of vaccine equipment, knowledge of healthcare workers and stock management practices were positively associated with vaccine cold chain management at 52.8%.

**Conclusion:**

Knowledge of vaccine handlers and stock management practices should be improved to enhance effective vaccine management. Besides, there is a need for the County Government of Vihiga to purchase sufficient WHO-recommended refrigerators.

## Background

The optimal potency of vaccines depends on the storage and handling practices at all levels. Vaccines are biological products that are sensitive to both heat and freezing; therefore, they need to be stored within recommended temperature ranges of between +2°C and +8°C in a cold chain system (World Health Organization, 2016). Child vaccination is among the most significant achievements in promoting child health and preventing infectious diseases. Immunisation programmes greatly depend on effective and efficient supply chain systems to transport, distribute and store vaccines and health commodities. This will in turn ensure that the right products are available at the right place, in the right condition and at the right time to provide efficient health services to the communities (Chiodini, 2014).

World Health Organization (n.d.) global data analysis on Effective Vaccine Management indicated that none of the regions meet the EVM standard of ≥80% in stock management and performance gets progressively weaker as vaccines flow through the supply chain (World Health Organization, 2020). One in just three countries within the (World Health Organization, n.d.) membership goes through stock-out of at least a single vaccine per month (Lydon et al., 2017). This represents a 33.3% stock-out level for one vaccine each month in the WHO member states. The same study continues elaborating that 39% of the stock-outs were a result of government delays in funding. Delays in the whole vaccine stock procurement process took 28% of the attribution while 18% was attributed to ineffective and poor stock management located at the country levels. Poor stock management within the global, regional and even local scanning of countries is a significant challenge for vaccine cold chain management (Lydon et al., 2017).

WHO created practice guidelines for different service levels that include vaccine monitoring, immunisation techniques, cold chain management and reporting systems (World Health Organization, 2018). These procedures when followed are bound to significantly improve vaccine storage and stock management in health facilities (Feyisa, 2021). Furthermore, it has been estimated that vaccine storage and stock management mainly require adequate and appropriate cold chain equipmentwithout which the vaccines lose their viability (Kumar & Gupta, 2020). Also, studies have shown that the knowledge and skills of the vaccine management staff are important to improve the cold chain management practices and so are the stock management practices (Chiodini, 2014; Kumar & Gupta, 2020).

Ineffective vaccine storage systems have been identified as one of the key reasons for vaccine stock-outs and loss of vaccine viability in many health facilities around the world (Lydon et al., 2017). Another study conducted in Iraq reported that poor stock management within the global, regional and even local scanning of countries is a significant challenge for vaccine cold chain management (Zayer & Chiad, 2020). Along the same line, studies conducted in South Africa (Chinwe Juliana Iwu et al., 2019) and Ethiopia (Feyisa, 2021) also attest to the fact that poor stock management is a significant challenge for vaccine cold chain management. These studies also indicated that low level of knowledge and skills regarding vaccine stock and storage management as well as misalignment of the stock and storage procedures have led to massive vaccine stock mismanagement.

In Kenya, significant stock-outs associated with poor stock management and low government funding among other factors were reported (Kanja et al., 2021). A study done to examine vaccine stock management and challenges in Nairobi County found that stock-outs were due to a lack of knowledge regarding vaccine stock management and inadequate physical, financial, human and technological resources related to vaccine storage management (Kanja et al., 2021). Nonetheless, these studies have been done in urban areas like Nairobi but not in semi-urban or rural areas like Vihiga County, and thus the present study is important and necessary as it contributes to the growing body of knowledge vaccine storage and stock management. It is expected that conducting a study in semi-urban and rural areas will help to identify any gaps that need to be addressed to improve the cold chain management and vaccine availability within the county, thus complying with the global standards. Therefore, this study assessed the status of the cold chain equipment, in Vihiga County, the stock management practices in place as well as knowledge of the personnel involved in the vaccine cold chain management.

## Methods

### Study area

The study was done in Vihiga County, which is one of the 47 counties in Kenya. The county has a population of 591,000. Within the county are 86 health facilities that offer immunisation services. The Ministry of Health in 2021 (Ministry of Health Kenya, 2021) noted that Vihiga is one of the counties that have the lowest vaccine coverage in Kenya at 79% compared to the county with the highest coverage which is Nairobi at 86%. Therefore, an examination of vaccine cold chain management in Vihiga is important.

### Research design, population and sampling

This was a descriptive cross-sectional design using a primary quantitative approach to assess the vaccine stock and storage management practices in Vihiga County, Kenya. The participants in this study were healthcare workers involved in handling and administration of vaccines from health facilities in Vihiga County. The census approach was adopted where all the 86 functional public health facilities were included in the study. At least one nurse in each health facility provides immunisation services. Therefore, one nurse who is regularly involved in immunisation services was purposively selected to respond to the questions. This gives a total of 86 participants.

### Data collection tools and procedures

The data were collected using researcher-administered questionnaire designed using the WHO-UNICEF Effective Vaccine Management (EVMA 2.0) Tool (World Health Organization, n.d.). In addition, an observational checklist was developed based on the WHO vaccine management assessment tool which was used to collect data on the status of the cold chain equipment and the vaccine management practices in the public health facilities. The data were collected in all immunising health facilities in Vihiga County from January to February 2023. The first author visited the selected facilities and explained the purpose and objectives of the study to all respondents. Those, who were selected and consented to participate, were interviewed by the first author and then they filled out the questionnaire accordingly. After that, the various indicators were inspected and the observation checklist was filled by the first author as per the findings of observations.

### Data analysis

Data analysis was done using Statistical Package for Social Sciences (SPSS) version 25. Variables, such as healthcare workers training on vaccine management, vaccine storage and stock management practices, were assessed. Descriptive statistics were used for variables on stock management practices including calculation of vaccine wastage rate, ordering and receiving of vaccines and on status of cold chain equipment including the availability of functional refrigerators and temperature monitoring devices and the availability of an alternative source of energy. In addition, a one-step linear regression analysis was used to determine the factors associated with vaccine cold chain management practices. Vaccine cold chain management practices were determined by computing the significant values from the descriptive analysis.

## Results

### Socio-demographic characteristics of the respondents

The findings from this study show that of the 86 immunising healthcare staff working in public health facilities in Vihiga County majority were females (84.9%). Furthermore, the majority had worked for the vaccine service between 2 and 10 years (63.9%). Also, the majority of the immunising healthcare workers had diploma certificates (93.0%). Additionally, the majority (65.2%) had received training in vaccine cold chain management, as indicated in Table 1.

**Table 1.** Socio-demographic characteristics of the study participants (*n* = 86).

Variable	Frequency	Percentage
Gender		
Male	13	15.1
Female	73	84.9
Experience in the vaccine service		
<2 years	16	19.8
2–5 years	29	33.7
6–10 years	26	30.2
>10 years	15	16.3
Level of education		
Certificate	2	2.3
Diploma	80	93.0
Bachelor’s degree	4	4.7
Training in vaccine cold chain management		
Yes	56	65.2
No	30	34.8

### Status of cold chain equipment in Vihiga County

Table 2 shows that at least one functional refrigerator in health facilities was available (70.9%). The fridge power supply was mainly sourced via electricity in 70.9% of the facilities and there was a functional temperature monitoring device in the refrigerator in the majority of facilities (70.9%). 57.0% of the facilities had discarded vaccines due to incorrect stock temperatures in the last six months. Furthermore, the results indicated that only 6% of the facilities had a responsible person for cold chain equipment preventive maintenance and only 4% of health facilities had power backup.

**Table 2.** Status of cold chain equipment (*n* = 86).

Variable	Frequency	Percentage
Availability of at least one functional refrigerator	61	70.9
Type of power supply for fridge		
Electricity as the main source	61	70.9
Solar as the main Source	0	0.0
N/A	25	29.1
Availability of functional temperature monitoring device in refrigerator	61	70.9
Type of temperature monitoring device		
Thermometer	0	0
Fridge tag	61	70.9
Not applicable	25	29.1
Discarded vaccines due to incorrect stock temperatures in the last six months?		
	49	57.0
Available person responsible for cold chain equipment preventive maintenance?		
	6	7.0
Availability of functional electricity backup	**4**	**4.7**

### Stock management practices in Vihiga County

The findings indicated that the majority of public health facilities visited have a vaccination micro plan (88.4%) and all facilities (100.0%) use standard vaccine requisition forms for ordering and receiving vaccines. 70.9% of health facilities do physical counts monthly. Furthermore, there is not quite a serious and proper stock of vaccines in the refrigerator (57.0%). Moreover, most facilities record vaccine and diluent quantities, batch number, expiry date and VVM status and also calculate vaccine wastage rate (70.9%). Table 3 provides more details.

**Table 3.** Stock management practices (*n* = 86).

Variable	Frequency	Percentage
The presence of a vaccination micro plan	76	88.4
Use standard vaccine requisition forms	86	100
Physical stock done monthly	61	70.9
Proper storage of vaccines in the refrigerator	49	57
Vaccine and diluents quantities, batch no and expiry date recorded		
	61	70.9
Calculation of vaccine wastage rate	61	70.9

### Knowledge of the healthcare workers on vaccine cold chain management practices

Table 4 shows that the immunising healthcare workers and handlers knew how to condition icepacks (88.4%) and prevent vaccine freezing during transport (88.4%). Most of the respondents (87.2%) disagreed that the opened multi-dose vial policy (MDVP) should be kept for the next immunisation sessions with visible labels. However, many did not know all the heat-, cold- and light-sensitive vaccines (57.0%) and only half of the participants knew the correct interpretation of the shake test (50.0%).

**Table 4.** Knowledge of healthcare workers on vaccine cold chain management practices (*n* = 86).

Variable	Frequency	Percentage
Knew how to condition icepacks		
	76	88.4
Opened multi-dose vial policy (MDVP) kept for the next immunisation sessions with visible labels		
	11	12.8
Knew temperature range for vaccines stored in the refrigerator		
Yes	86	100.0
Naming all heat-sensitive vaccines	36	41.9
Naming all cold-sensitive vaccines	37	43.0
Naming all light-sensitive vaccines	37	43.0
Knowing how to read and interpret VVM	86	100.0
HF following the first expiry first out (FEFO) in practice		
	61	70.9
Knowing the correct interpretation of the shake test		
	43	50.0

### Factors associated with vaccine cold chain management practices

To assess the extent to which the status of vaccine cold chain equipment, knowledge of immunising healthcare workers and vaccine handlers and stock management practices influence vaccine cold chain management practices at the public health facilities in Vihiga County, regression analysis was done by computing the significant values from the descriptive analysis. The results are shown in Table 5. Status of vaccine equipment, knowledge of healthcare workers and stock management practices were positively related to vaccine cold chain management. These factors altogether explained 52.8% of the variance in vaccine cold chain management.

**Table 5.** Predictors of vaccine cold chain management practices.

Model	Standardized coefficients beta	*T*	Sig.
Constant	.228	0.093	.908
Status of vaccine equipment	.536	2.501	.001
Knowledge of healthcare workers	.501	2.522	.001
Stock management practices	.372	3.60	.000
Adjusted *R* square	.528		

## Discussion

This study assessed the vaccine storage and stock management practices in Vihiga County, Kenya. The study revealed that the majority of health facilities have at least one functional refrigerator. The findings of this study were slightly lower than a similar survey conducted in Nigeria, where 92.7% of the health facilities assessed had at least one functional refrigerator (Ogboghodo et al., 2017). Regarding the contingency plan, only very few had power back up which is lower than the findings of a study done in Ethiopia which had 31% (Bogale et al., 2019) and Nigeria 30.6% (Ogboghodo et al., 2017). The absence of functional refrigerators in some health facilities and the lack of alternative sources of power for refrigerators may be due to a lack of funds and a lack of policy to ensure effective vaccine management at health facilities. If not well-addressed, this observation may cause vaccine wastage and increased missed opportunities in immunisation.

Adherence to a routine planned preventive maintenance of refrigerators in Vihiga County Kenya was very low. The low percentage was also reported in Ethiopia at 14% (Feyisa, 2021). This may be due to a shortage of biomedical staff in Vihiga County. According to the recommended vaccine storage temperature range (+2°C to +8°C), many health facilities had a functional temperature monitoring device in the refrigerator, which is higher than those reported in Cameroon which had 32.7% functioning thermometers (Ateudjieu et al., 2013). Also, the majority of respondents recorded temperature regularly. This reported practice is higher than the study conducted in Ethiopian health facilities at 51.2% (Feyisa, 2021) and Cameroon at 40.7% (Ateudjieu et al., 2013). This may be due to the unavailability of a functional temperature monitoring tool in health facilities in different countries, and if not addressed can compromise the safety and potency of vaccines.

The overall stock management practice is effective in public health facilities in Vihiga County except for the lack of proper storage of vaccines in the refrigerator probably due to the lack of refrigerators in dispensaries. The current findings are slightly lower than those reported in Ethiopia at 58.3% (Bogale et al., 2019). The WHO (World Health Organisation, 2018) offers a vaccine stock management procedure and guideline rule book. In the guideline, WHO promotes the use of a vaccine stock management system that allocates and tracks the whole process of the vaccine from resourcing to final dispensing and evaluation.

The current result shows that this is generally done within the public health facilities. The results agree with the existing literature which revealed that effective vaccine stock management ensures that the vaccines are kept and maintained within acceptable standards and the vaccines are continuously and adequately available at the service delivery stage (Duttagupta et al., 2017). The robust use of a Vaccine Vial Monitor (VVM) in Vihiga County also agrees with the literature in a study done in Albania (Kartoglu et al., 2020) that observed the VVM has remained, consistently, the best tool that health facilities have used in Europe to monitor and maintain effective stock and vaccine potency management. This is because VVM provides a reliable signal to warn health workers whether the vaccine has been affected by heat and whether it can be used.

The vaccine cold chain handlers were knowledgeable regarding many elements of vaccine cold chain management. The current results are slightly higher than those reported in Ethiopian health facilities at 82.0% (Feyisa, 2021) and Nigeria at 86.3% (Ogboghodo et al., 2017). However, the current findings revealed that health workers have limited knowledge of heat-sensitive, light-sensitive and cold-sensitive vaccines. This contrasts the findings of a study conducted in Ethiopia where 96.7% of vaccine handlers have good knowledge of this subject matter (Bogale et al., 2019). The reported lack of knowledge in the current study may be due to a lack of training on cold chain management to enable effective and proper storage of vaccines in the refrigerators which can lead to loss of potency of vaccines. To address this gap training, mentorship and support supervision should be done frequently. The training can range from temperature monitoring techniques including how to properly calibrate thermometers, interpret temperature readings, respond to fluctuations, regular equipment inspections, cleaning protocols and preventive maintenance measures. This in turn can effectively maintain optimal storage conditions and prevent vaccine spoilage. Furthermore, during this training, participants can learn about the various stages of the cold chain, from manufacturing to distribution to administration and understand the significance of maintaining consistent temperature control at each stage to ensure vaccine efficacy. By equipping personnel with this knowledge and skills, the risk of storage failures and vaccine wastage can be minimised.

The current findings showed that vaccine cold chain management was predicted by the status of vaccine cold chain equipment, knowledge of healthcare workers and stock management practices which is slightly higher compared to the study conducted in Ethiopian health facilities, where the same factors predicted vaccine cold chain management at 48% (Gebretnsae et al., 2022). This indicates that these factors play a significant role and should be addressed to improve cold chain management.

## Strength and limitations

The strength of this study is based on the use of more than one instrument to collect data thus enhancing the reliability of the results. It reports the extent to which the status of vaccine cold chain equipment, knowledge of healthcare workers and stock management practices predict the vaccine cold chain management, thus, providing information on how addressing these factors is a key to effective vaccine management.

The findings in this study were limited to public health facilities within one county with a small sample size out of the 47 counties in Kenya, therefore, cannot be generalised for the whole country. The research was largely quantitative and thus mainly determined the ‘what’ and therefore, did not determine the why aspects of vaccine cold chain management. Further research should be done to engage with an in-depth qualitative study to determine the why aspects of vaccine cold chain management.

## Conclusion

This study assessed the vaccine storage and stock management practices in Vihiga County, Kenya. The findings of this study revealed that the majority of public health facilities’ vaccine storage conditions and stock management practices were in line with the WHO guidelines. The majority of immunising healthcare workers knew how to condition icepacks 88.4%; however, 57.0% did not know all the heat-, cold- and light-sensitive vaccines. Status of vaccine equipment, knowledge of healthcare workers and stock management practices were positively associated with vaccine cold chain management at 52.8%. In conclusion, knowledge of vaccine handlers and stock management practices should be improved to enhance effective vaccine management.

## Ethics approval and consent to participate

Approval was obtained from Maseno University Scientific and Ethics Review Committee [MURSERC/01158/22], National Commission for Science Technology and Innovation (NACOSTI) (Research License Reference Number NACOSTI/P/23/22740). At the county level, ethical approval was sought from the office of the Director of Health and Informed consent from all voluntary study participants.

## Data Availability

Data used in this study are available upon a reasonable request from the first author.
